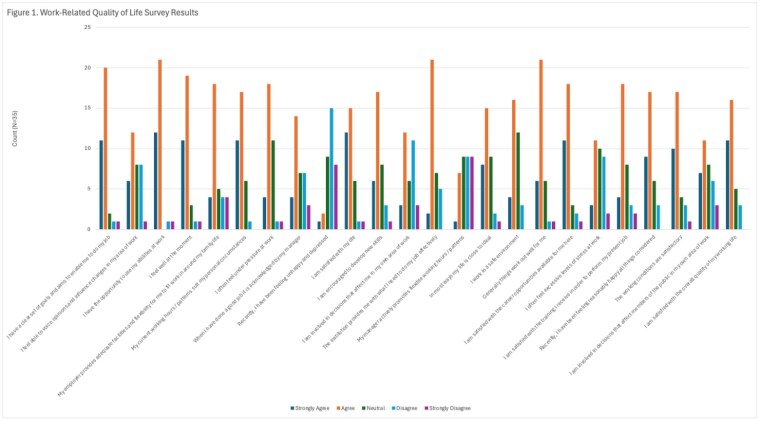# 100 Work-Related Quality of Life in Firefighters

**DOI:** 10.1093/jbcr/iraf019.100

**Published:** 2025-04-01

**Authors:** Micayla Kotarski, Emily Beutler, Callie Thompson

**Affiliations:** University of Utah Health Burn Center; University of Utah Health; University of Utah Health

## Abstract

**Introduction:**

Firefighters (FFs) are the foundation of community safety in the United States. These first responders play a critical role in the care of the burn patients. The nature of the work requires these individuals to be exposed to repetitive secondary trauma in high-stress and high-risk environments. Ensuring the long-term comprehensive health of the FF workforce will be critical in the continued successful delivery of burn care. We sought to understand the Work-Related Quality of Life (WRQoL) in local FFs to develop future interventions to improve WRQoL and resilience.

**Methods:**

After IRB approval, we sent an anonymous survey via email to all members of our local firefighter union in August 2024. We collected minimal demographic data and inquired about WRQoL, feelings of safety, and intention to leave in the 3 months (mo.) prior to the survey.

**Results:**

Demographics were as follows: 63% FF, 28% Paramedic, and 9% Management, 77% of respondents had more than 6 years on the job and 23% had 1-5 years on the job. Responses to the WRQoL Scale are in Figure 1. Forty-six percent report having an event in the last 3 mo. that they thought about more than they’d like or are still working through. Forty-three percent of respondents never think about leaving their current job or the profession entirely.

**Conclusions:**

Our results indicate that nearly half of this experienced cohort of firefighters report being burdened by recent events which highlights the potential utility of an intervention to help them manage those thoughts and feelings. However, despite their WRQoL indicating areas for improvement regarding involvement in decisions that impact them, more flexibility in working hours, and acknowledgement of their good work; 76% are satisfied with their overall quality of working life and a near-majority never think about leaving the job.

**Applicability of Research to Practice:**

Understanding the WRQoL of firefighters is imperative for future interventions to reduce burnout and increase resilience in this critical workforce.

**Funding for the Study:**

N/A